# The Effect of Videoconferencing on Second-Language Learning: A Meta-Analysis

**DOI:** 10.3390/bs12060169

**Published:** 2022-05-30

**Authors:** Li-Tang Yu

**Affiliations:** Department of English Instruction, College of Education, National Tsing Hua University, Hsinchu 300193, Taiwan; ltyu@mx.nthu.edu.tw

**Keywords:** meta-analysis, synchronous computer-mediated communication, videoconferencing, second-language learning

## Abstract

To mitigate the unexpected closure of educational institutions during the COVID-19 pandemic, e-learning has become a practical alternative to conventional face-to-face instruction. Videoconferencing, a synchronous computer-mediated communication (SCMC) approach, has been adopted as a venue to continue student learning activities. However, in the field of second-language (L2) education, videoconferencing had already been integrated into learning tasks, enabling L2 learners to have more opportunities to access authentic linguistic input and participate in interactions with more proficient users or native speakers of the target languages. Research has reported the pedagogical benefits of learners’ L2 achievement that are provided by videoconferencing, whereas some studies have reached a different conclusion. To further ascertain the effectiveness of videoconferencing in L2 learning, meta-analysis can be used to provide statistical evidence of the significance of study results, which serves as a useful reference for the application of videoconferencing to current e-learning practices. Thus, systematic meta-analysis was used in this study to synthesize the findings from experimental and quasi-experimental research into the effectiveness of videoconferencing for L2 learning. Videoconferencing approaches led to positive, medium overall effects in control/experimental group comparisons (*g* = 0.35, *p* < 0.5) on the L2 language development of listening and speaking abilities. However, this conclusion is based on five studies and, thus, needs to be treated cautiously. The implications of the findings and suggestions for future studies are discussed.

## 1. Introduction

The unexpected breadth of the COVID-19 pandemic forced the closure of educational institutions to minimize people’s interaction, reducing the transmission of the virus. To continue learning in such circumstances, teachers and students shifted from conventional face-to-face instruction to e-learning, resorting to the use of synchronous computer-mediated communication (SCMC) to continue pedagogical activities. Many videoconferencing tools, such as Zoom, Google Meet, and Webex, to name a few, were thus developed to fulfill people’s needs to digitally interact/communicate with each other [1].

While some teachers and learners might have been busy shifting to e-learning and handling the hustle and bustle of SCMC tasks, second-language (L2) teachers and learners were more likely to acclimate to this new model. The adoption of SCMC in the field of L2 teaching and learning has become increasingly popular in recent years. The pedagogical benefits regarding learners’ L2 achievement that is provided by videoconferencing have been reported in various studies [2,3,4]. To further ascertain the effectiveness of videoconferencing in L2 learning, meta-analysis can be used to provide statistical evidence of the significance of study results. This information can be a useful reference for the application of videoconferencing to current e-learning practices.

SCMC can be undertaken in different modes, such as text-based, audio-based, and video-based modes, or some combination of them. Its effect on L2 learning has been widely researched [5,6]. During SCMC activities, L2 learners are able to receive more authentic linguistic input and prompt feedback, and they have more language-use opportunities to interact with native speakers of their target language or more competent L2 users, such as peers or teachers [4,7,8]. Moreover, L2 learners can participate in SCMC without the constraint of brick-and-mortar language-class locations. This type of online learning is supported by interaction [9] and sociocultural [10] approaches, as affirmed by relevant, empirical studies [11,12,13]. During SCMC activities, L2 learners engage in peer-to-peer or expert–novice interactions and receive feedback from others. The feedback enables learners to become aware of their linguistic deficiencies and obtain the scaffolding to re-organize their linguistic mental processes, which is a crucial element in L2 learning, according to both interactionist and socio-cultural theories. The positive effect of SCMC on L2 learning has been acknowledged by recent meta-analytical studies [14,15,16].

Among the various modes of SCMC, videoconferencing with multimodal features promotes interaction and allows users to communicate simultaneously with each other through audio, video, text, or combinations thereof [17,18]. With the current advances in information technology and Internet bandwidth, videoconferencing has been attracting attention from L2 researchers and educators. Its application in L2 learning has become increasingly prevalent. Research interest in using videoconferencing in L2 acquisition is growing continuously. The multimodality affordance of videoconferencing creates dynamic meaning-making processes, granting users the ability to receive and deliver meanings with more than one representation mode, such as linguistic, visual, and gestural elements, which fulfills the nature of communication. Multimodality affords a multitude of possibilities for L2 learners to negotiate meaning, express themselves better, perform various discourse functions, and interact with others effectively [19,20]. Thus, it is of importance to evaluate the strength of evidence derived from such empirical studies. Regardless of some systematic reviews and meta-analysis studies on computer-mediated communication [14,15,21] and, furthermore, some conducted specifically on SCMC [16], these studies do not explicitly discuss how the multimodality of videoconferencing contributes to L2 development. To fill this gap, the current study aims to systematically summarize and analyze the effect of videoconferencing on the development of L2 learning. Close attention to the relevant studies is paid, aiming to “identify patterns among the study results, and develop a more conclusive estimate of the magnitude of the effect of a particular variable” [22] (p. 104).

## 2. Literature Review

### 2.1. Using Videoconferencing for L2 Learning Purposes

The application of videoconferencing for a wide range of L2-learning purposes has been growing in popularity. It allows real-time communication between users and builds social presence through its multimedia features [23]. More and more studies have been conducted to examine the effect of videoconferencing tasks on promoting L2 learners’ development of language abilities and other affective aspects (e.g., motivation, confidence, and interest). Several pedagogical benefits that L2 learners can obtain through videoconferencing activities have been identified.

Firstly, videoconferencing promotes L2-learner language skills. For example, learner speaking skills can be improved. Lee [4] found that Spanish learners actively participated in videoconferencing tasks and improved their speaking abilities when they undertook interactive activities with native Spanish speakers, based on the recorded conversations and the interview responses. Yen, Hou, and Chang [24] demonstrated that the combination of asynchronous text discussion and synchronous videoconferencing interaction was beneficial in the development of English writing skills and speaking abilities for L2 learners of English, as evidenced by their significant progress in the speaking post-test outcome. Tecedor and Campos-Dintrans [25] reported that Spanish university learners improved their fluency and complexity (referring to their ability to use a wide array of language structures) in presentational tasks, as well as fluency, accuracy, and complexity in interpersonal tasks, after participating in videoconferencing activities. Saito and Akiyama [26] also found significant gains in the spontaneous English production of university students in Japan when they undertook video-based interaction with competent English users. In addition to improved speaking abilities, a study showed that Korean learners at a university in the United States who worked with their assigned videoconferencing interlocutors in Korea reported, via surveys, journals, and interviews, an improvement in their listening abilities [27]. The positive effect of videoconferencing on listening-comprehension ability was also confirmed in Levak and Son’s study [2], as well as that of Saito and Akiyama [6,26], both of which measured participant listening abilities in listening tests and found improvements in the test outcome. Aside from the positive gains in speaking and listening skills, L2 learners could improve their vocabulary knowledge in videoconferencing tasks. Yanguas [28] confirmed that the L2 vocabulary knowledge of Spanish college learners was fostered through videoconferencing interaction since their performance in the production and recognition lexical tests was significantly enhanced.

Secondly, videoconferencing tasks could enhance language-learners’ intercultural awareness, which is “a conscious understanding of the role culturally based forms, practices, and frames of understanding can have in intercultural communication, and an ability to put these conceptions into practice in a flexible and context-specific manner in real-time communication” [29] (p. 66). Intercultural interaction via videoconferencing enables learners to become sensitive to the target culture of their videoconferencing, better recognize various aspects of the target-language culture, and, more critically, reflect upon it [30,31]. Moreover, learners can gain more intercultural communicative abilities, such as openness to and curiosity regarding their videoconferencing partners’ culture, making a connection between other people’s cultures and their own, and motivating them to share their own culture with others [32,33,34].

Finally, via videoconferencing interaction, L2 learners can become more confident, motivated in speaking their target language, and interested in the learning content. Lee et al. [35] embedded videoconferencing into their English-as-an-international-language (EIL) course and arranged for learners to interact with EIL experts in inner-, outer-, and expanding-circle countries. They found that their learners had positive attitudes toward the course. Jauregi and colleagues [36] showed that synchronous videoconference collaborative learning between native and non-native speakers effectively improved L2 learner motivation. Videoconferencing activities could promote learners’ language confidence. Phillips [5] demonstrated that videoconferencing could be beneficial in L2 students with different proficiency levels. After videoconferencing with native speakers of French, those students with lower proficiency levels gained more confidence in speaking French, and those with higher proficiency levels became highly engaged in interacting with their videoconferencing interlocutors in French. The same conclusions were confirmed by Pritchard et al. [37] and Yu [38], that learners who participated in videoconferencing activities gained more speaking confidence.

However, not all empirical studies evidenced better L2-learning outcomes resulting from videoconferencing. For example, Rassaei [39] stated that participants’ speaking performance was comparable in both videoconferencing and face-to-face contexts. Yang and Chang [40] found that Taiwanese learners of English did not improve their oral skills at the end of videoconferencing sessions. In Sooryah and Soundarya’s study [41], L2 learners expressed their difficulties when listening to lectures in videoconferencing settings. Terantino [42] made the observation that L2 learners had similar levels of speaking anxiety in both videoconferencing oral tests and face-to-face tests. Furthermore, Yu [38] found that participants felt more anxious during videoconferencing tasks than in conventional language classes. Thus, there is a need to examine the relevant data from studies for the purpose of exploring, with rigorous statistical techniques, the efficacy of videoconferencing in the development of L2 competence. In this sense, a meta-analysis provides a way of synthesizing the results of studies and determining the estimated effect size, which is an objective evaluation of the integrated quantitative evidence [43].

### 2.2. Systematic Review and Meta-Analyses of Synchronous Computer-Mediated Communication for L2 Learning

Computer-mediated communication for various second-language acquisition (SLA) purposes has been one of the most widely studied research fields [21]. A plethora of studies has focused on the impact of different forms (asynchronous and synchronous) and modes (text-based, voiced-based, video-based, or some combination of them) for different learning purposes. In order to rigorously examine the effects reported in various research works on a particular topic, systematic reviews and meta-analyses are feasible, effective approaches to identifying patterns in a range of research results and predicting the impact of specific variables [44]. The application of systematic reviews enables researchers to methodically conduct a secondary review of a large body of previous studies [45]. Additionally, meta-analyses can offer quantitative, objective data to researchers by means of statistically examining individual studies on a specific topic and, thus, averaging the effects reported across the studies [46].

To the researcher’s knowledge, there has been only one systematic review [21] and three meta-analyses [14,15,16] on L2 computer-mediated communication, as displayed in Table 1. These studies provide informative insights into our understanding of the application of computer-mediated communication to L2 learning. However, there are some limitations. Firstly, the research work included in these studies was not contemporary. The most recent research that was analyzed was published in 2012. Secondly, the focus of the systematic review and meta-analyses does not provide a comprehensive picture of a specific mode of SCMC for L2-learning purposes, namely, video-based interaction. As empirical studies have attested, the effect of videoconferencing on the development of L2 abilities is distinct from that of other modes, such as text-based or voice-based communication. For example, Angelova and Zhao [47] examined the dyads of native and non-native speakers in a collaborative task, through text-based discussion and videoconferencing. They found that language learners had more exposure to their target language through different forms of computer-mediated communication (CMC). Both CMC modes widened the exposure of language learners to native speakers who would otherwise be inaccessible. However, videoconferencing provided learners with more audio and visual clues by which to understand the target language and culture. Moreover, Cohen and Wigham [48] examined the impact of an audio-based interaction mode and a video-based interaction mode on the vocabulary expression of nine upper-intermediate learners of English. In contrast to audioconferencing, the learners gave more semantic information through speech and gesture to their interlocutors in videoconferencing with a combination of visual and audio expressions. These findings emphasize the specific benefits afforded by videoconferencing for L2 learning. Thus, the current research aims to expand the previous systematic review and meta-analyses and overcome their limitations. Published work related to videoconferencing for L2 learning was included so as to shed light on the effectiveness of such a pedagogical intervention in the development of L2 abilities.

As research on the use of videoconferencing for L2-learning purposes has grown continuously in recent years, a meta-analysis of these empirical studies is timely. It is expected to broaden our knowledge of the field of SCMC and elucidate our understanding of this prevalent pedagogical intervention.

One overarching research question guided the study: What is the overall effect of videoconferencing on L2 learning?

## 3. Methodology

Four steps, as suggested by Cooper [44], were taken in the current meta-analytical study: (1) searching for relevant research papers; (2) setting inclusion criteria for the retrieved studies; (3) coding the research papers; (4) calculating the effect size of the research papers.

### 3.1. Searching for Relevant Research Papers

With reference to the searching strategies adopted in previous systematic review and meta-analysis work [14,15,16,21,49,50], several electronic databases were consulted, such as the Web of Science (WOS), Scopus, ERIC, LLBA, JSTOR, and ProQuest-Education. Using multiple sources when searching for studies is an effective strategy [51]. The search keywords included computer-assisted language learning, computer-mediated communication, synchronous, computer-assisted instruction, online interaction, and videoconference/videoconferencing. Other keywords were used in the search process: EFL, ESL, second language, second-language acquisition/learning, and foreign language.

### 3.2. Inclusion Criteria for Retrieved Studies

With reference to the inclusion and exclusion criteria applied in previous systematic review research [16,21,50,52], the following criteria were modified to fit the purpose of the current study, in order to examine the eligibility of the searched studies.

1. The study should present data from 2000 to 2021.
2. The study should be written in English.
3. The study should be published in an academic peer-reviewed journal.
4. The study should examine the effect of videoconferencing in a way that is either exclusive or in conjunction with other modes of computer-mediated communication on various aspects of L2 learning. In other words, participants in the study should use videoconferencing to develop their L2 skills.
5. The study should be empirical, adopting a quasi-experimental (participants are non-randomly assigned to research groups [53]) or experimental design (subjects are randomly assigned to different research groups [53]) with at least one experimental group (videoconferencing tasks) and one control/comparison group (traditional teaching or computer-mediated activities other than videoconferencing tasks) and providing quantifiable data for the measures.
6. Participants should be second-/foreign-language learners.

### 3.3. Coding Research Papers

The research papers searched in the present study followed the PRISMA guidelines. In the first search for relevant research papers, 184 articles were found. In the second search, duplicate articles that were found in the various academic research databases were eliminated. Consequently, 89 studies remained. In the third round of the paper search, the six criteria described above were applied to screen the studies; after which, only 11 studies remained. In the final search, statistical information (i.e., means, standard deviations, and group sizes) was required for the calculation of the effect size, thus excluding six studies. In the end, five studies formed the body of the meta-analysis.

With reference to a previous study [16], a coding scheme was drafted. As shown in Table 2, the application of codes led to an understanding of the nature of videoconferencing interaction, in terms of their contextual variables and research design.

The researcher and his research colleagues, with substantial training in research methodology, formed a team to code the retrieved studies. Each of the team members was the primary coder to code assigned studies independently, and then coded each other’s studies as a secondary coder. The inter-coder reliability was examined by checking the agreement between codes for the same study given by different coders. The agreement ratio was high (95.45%). The inconsistencies were resolved through discussion.

### 3.4. Calculating the Effect Size of the Research Papers

To present the overall efficacy of video-based computer-mediated communication on L2 learning, the estimation of Hedge’s *g* [54] was calculated. To measure Hedge’s *g* values, information from individual retrieved studies, such as means, standard deviations, and sample sizes, was recorded. Based on Cohen’s criteria [55], the magnitude of the effect of videoconferencing on L2 learning was interpreted as follows: small effect (less than 0.20); medium effect (between 0.21 and 0.80); and large effect (greater than 0.80). The Comprehensive Meta-Analysis software program version 3 (Englewood, NJ, U.S.A.) [56] was adopted for the statistical meta-analysis. The program creates an interface that is similar to Excel sheets, enabling users to key in the study data for analysis. Various data can be accommodated in the program, including independent subgroups, sample sizes, outcome means, and standard deviations, to name a few. Moreover, the program can assess the heterogeneity of the data and evaluate the publication bias derived from collected studies. It is recognized as one of the most sophisticated software programs for meta-analysis [57].

## 4. Results

### 4.1. Descriptive Information

A brief overview of the five selected studies is presented in Table 3. Five studies were coded based on the study features in Table 2. All studies except that of Alshahrani [23] were indexed in SSCI, whereas Alshahrani [23] was indexed in SCOPUS. The studies were published from 2016 to 2019. The sample sizes ranged from 30 to 57. All the participants in the studies were university students. In most studies, the target language was English, and participants studied in English-as-a-foreign-language contexts, while only one study [25] explored Spanish learning in the foreign-language context. Finally, the proficiency level of the participants’ target languages varied. It was not reported consistently. One study stated having participants at the beginning-proficiency level [25], one had intermediate-level participants [39], and one had participants whose proficiency ranged from pre-intermediate to intermediate levels [23]. The studies conducted by Saito and Akiyama [6,26] merely stated that their participants had learned the target language for six years.

Two main language features were investigated in these studies. Four out of five primary studies were concerned with speaking performance [23,25,26,39]; there was only one study that focused on listening comprehension [6]. This research trend, with a predominant focus on L2 speaking skills, was in line with the findings of Lin et al.’s systematic review [16] on text-based SCMC, that the majority of analyzed studies were concerned with oral skills. A variety of issues was explored in the five primary studies. The speaking performance in these studies was further categorized into different aspects, such as fluency, accuracy, pronunciation, vocabulary, and communication approaches (question types, strategies, and recasts). The listening comprehension targeted the understanding of spoken discourses.

The treatment duration of the five primary studies varied. The treatment length of the five primary studies ranged from 10 days to 12 weeks. Rassaei’s study [39] was conducted for 10 days. Two studies spent 10 weeks completing their intervention [6,26]. Finally, Alshahrani [23] and Tecedor and Campos-Dintrans [25] spent 12 weeks on their research projects. As for how much time was spent on the videoconferencing context in the five studies, a variety of activity durations was identified, ranging from 14 min to one hour each time. For example, the videoconferencing activity in Rassaei’s study [39] was brief, at 14 min each session for 10 days. Other studies [23,25] were intensive, having two or three sessions per week. The remaining two [6,26] involved one session per week.

In terms of group division, the five studies used different grouping strategies. Three studies assigned their participants to one-on-one interaction in their videoconferencing tasks, either with teachers with native-like proficiency [39] or native speakers of the target language [6,26]. Tecedor and Campos-Dintrans [25] paired up learners to converse with each other. Lastly, Alshahrani [23] assigned all students in the experimental group to a single videoconferencing group that would videoconference with native speakers of English. With regard to the counterpart for the videoconferencing-effect comparison in each study, three studies adopted conventional face-to-face instruction [23,25,39]. Tecedor and Campos-Dintrans [25] had another comparison group that used asynchronous audio recording. Rassaei [39] and Saito and Akiyama [6,26] had a control group that had no interaction with others, such as studying learning materials alone and completing grammar and lexical practice tests.

With only five studies meeting the analysis criteria, it is feasible to present individual studies to deepen our understanding of how the effect of videoconferencing on L2 speaking and listening was investigated. To begin with studies on speaking competence, Alshahrani [23] adopted a pre–post speaking test design to examine the development of speaking proficiency. It was found that despite no difference in speaking performance being found in the pretest, the videoconferencing group significantly outperformed the non-videoconferencing group in terms of their speaking skills in the post-test. Next, Rassaei [39] investigated participants’ performance in speaking production tasks (i.e., to correct the use of articles in their expression) and error correction tasks (i.e., to identify the misuse of articles in English production) in the pretest and the post-test. The findings showed that either face-to-face or videoconferencing converse was equally effective for enhancing L2 speaking development. Moreover, Saito and Akiyama [26] measured the videoconferencing and non-videoconferencing groups’ speech production of timed picture descriptions. The finding showed that learners in the videoconferencing group achieved better comprehensibility of speech production than the non-videoconferencing group at the end of the study. Finally, Tecedor and Campos-Dintrans [25] examined beginning-level Spanish learners’ speaking performance by implementing communicative activities. The participants in videoconferencing (N = 16), voice recording (*n* = 14), and face-to-face (*n* = 18) modes made presentations and undertook peer-to-peer role-play activities. Researchers found that all modalities contributed to learners’ complexity and fluency in presentational tasks, as well as to fluency in role-play tasks, whereas the videoconferencing group did better with regard to complexity and accuracy in role-play tasks. Thus, it is evidenced that videoconferencing was an effective means of developing speaking competence.

In terms of listening skills, Saito and Akiyama [6] investigated the effect of different types of instruction (communicative discussions via videoconferencing (*n* = 15) and regular vocabulary and grammar practices (*n* = 15)) on the listening comprehension of Japanese EFL learners. They adopted TOEIC as a means of assessment to examine the participants’ pre-/post-test performance development. Despite both groups exhibiting improvement in listening performance, researchers reported that more progress was found in the videoconferencing group.

### 4.2. Overall Effect Size

To answer the overarching research question about the overall efficacy of videoconferencing in L2 learning, information such as Hedge’s *g*, standard error, and the confidence interval for effect size is presented in Table 4. As seen in Table 4, the Q statistics and *I*^2^ statistics demonstrated that the effect sizes did not reach a statistically significant difference, so the fixed-effects model was used to pool the data. This also suggests that no further moderator analysis is needed.

Multiple comparisons for the effect-size calculation out of five studies were conducted. As shown in Table 4, the effect sizes ranged from 0.11 to 0.82. Among the effect-size values, all five were positive (one small, three mediums, and one large). The weighted mean effect size across the five studies was 0.35 (medium, *p* < 0.05). The 95% confidence interval ranged from 0.06 to 0.65, which excluded zero. Thus, the use of videoconferencing reached statistical significance in L2 learning, displaying a positive, medium effect size. This result is consistent with those of other meta-analysis studies on the effect of general CMC or text-based SCMC on learners’ SLA [14,15,16]. This suggests that the effect of videoconferencing on L2 learning, especially in terms of speaking and listening skills, outperformed that of non-videoconferencing. In other words, L2 learners in videoconferencing contexts could more effectively enhance their L2 speaking and listening skills compared to those in non-videoconferencing contexts.

Moreover, the result of the Q statistic (*Q* = 2.83, *p* > 0.05) provided evidence that heterogeneity in effect sizes did not exist among the five studies. The *I*^2^ statistic, to establish the proportion of heterogeneity to total variance across the observed studies, was administered to analyze the extent of inconsistency across these findings. Its resulting index was viewed as low, based on the benchmark suggested by Higgins et al. [58]. Therefore, there is no need to run moderator analyses.

### 4.3. Evaluation of the Publication Bias

**Figure 1:**
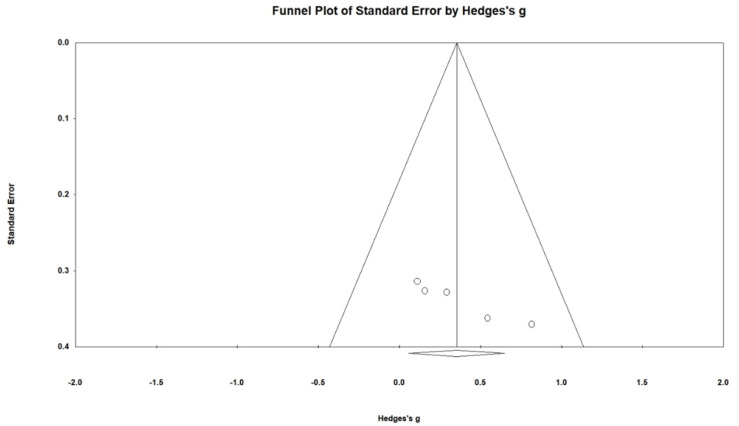
Funnel plot evaluating publication bias regarding L2 development.

The publication bias results indicate that studies reporting positive findings, submitted to and being accepted for publication in peer-reviewed journals, appeared more often than studies reporting negative or null findings [59]. To detect publication bias, two approaches were adopted. First of all, a simple visual inspection method, the funnel plot, was used. This is a scatterplot that presents the distribution of effect sizes. In the plot, the horizontal axis presents the range of effect estimates and the vertical axis shows the extent of the standard errors of the effect sizes. The distribution of effect sizes will appear symmetrical if there is no publication bias so that the plot will look like a funnel [60]. As demonstrated in Figure 1, the smaller number of studies created variance and were scattered across a range of values. To further confirm the presence of publication bias, Egger’s regression test [61,62] was thus adopted. Statistical evidence provided from Egger’s test analysis (*t* = 6.42; *p* < 0.01) ascertained the presence of publication bias.

## 5. Discussion and Conclusions

The goal of this meta-analysis is to systematically examine empirical studies that used videoconferencing, a type of SCMC activity, for L2 learning. A calculation was conducted to measure the overall effect size and to evaluate the efficacy of such applications to SLA. After careful screening, five studies that adopted quasi-experimental or experimental between-group comparison-design research were selected. It was found that the use of videoconferencing on SLA had a positive, medium effect on language learning (*g* = 0.35), and its effect was statistically evidenced (*p* < 0.05). The analysis outcome confirms Guichon’s assertion [8] and corroborates the previous meta-analytical literature [14,15,16], suggesting that L2 learners seem to perform better in videoconferencing contexts than in non-videoconferencing contexts, specifically in the development of listening and speaking skills. This finding could possibly be attributed to certain features of videoconferencing: firstly, the multimodality afforded by videoconferencing enables users to source a variety of inputs, such as audios, videos, texts, and images, and to process information through auditory and visual channels, which is akin to spontaneous communication in face-to-face settings [63]. Secondly, during videoconferencing, users can see each other. This mutual gaze helps to co-construct emotional experiences among users [64]. Finally, the high level of naturalness in videoconferencing communication is similar to face-to-face talk, fulfilling the criteria of the media naturalness theory, which comprise:

“(1) co-location in a common physical space, (2) synchronicity level that allows immediate and spontaneous reaction to stimuli, (3) the possibility to identify and transmit facial expressions, (4) the possibility to identify and transmit body language, and (5) the possibility to receive and transmit natural speech” [65] (paragraph 3).

These features are conducive to creating an interactive atmosphere, which is the nature of contemporary L2-teaching methodology, the communicative approach, a second-/foreign-language teaching method dedicated to promoting learner communication competence through actual language use in communication tasks, highlighting authenticity and meaningfulness via mutual interaction [66]. Furthermore, when e-communication is more natural, participants will experience fewer cognitive issues, communication ambiguity will be reduced, and physiological arousal will be increased because the human brain’s circuitry is hard-wired to process information in a “co-located and synchronous manner” [67] (p. 121). The videoconferencing also enables its users to create a virtual co-presence, as in a sense of being together with others, involving psychological engagement between the interlocutors [68]. Although the users are located in different places, they are all virtually present in each other’s physical space. As corroborated by Develotte et al. [69], videoconferencing “creates presence at a distance, installs an obvious connection between the participants and, furthermore, develops the quality of the pedagogical relationship (p. 309)”, allowing users to establish connections more easily between each other and to feel closer to each other [70]. Learners can have a sense of belonging and security in this learning community, which facilitates their learning [66]. Thus, videoconferencing could be viewed as a valid alternative to conventional brick-and-mortar instructional settings, especially when educational institutes were forced to shut down due to the COVID-19 pandemic. However, a caveat needs to be made. The presence of publication bias was detected in the current literature, suggesting that the included studies might not be representative of all studies conducted on this topic. The overall effect size of the available studies might be larger than if all relevant studies were included [71].

Our finding has implications for future videoconferencing-related research and practices. To begin with, it is confirmed that L2 learning in videoconferencing settings, especially regarding the promotion of speaking and listening skills, is more effective than in non-videoconferencing contexts. Hence, more pedagogical L2-learning tasks via videoconferencing could be encouraged. Moreover, the primary studies analyzed in the current work predominantly focused on tertiary-level students, their conversation abilities (listening and speaking), and English as a foreign language. This impedes our understanding of the extent to which L2 learners at various academic levels could benefit from videoconferencing in various contexts. In particular, learners with different proficiency levels would approach language learning with various strategies, behaviors, and characteristics [72,73]. Therefore, more attempts should be made to explore the potential of videoconferencing for the development of different language skills in various contexts for a wide range of L2 learners. Consequently, L2 researchers, instructors, and learners could be better informed about the efficacy of videoconferencing on the development of L2 abilities.

The limitations of this meta-analytical work must be acknowledged. Firstly, a small number of studies was collected in the analysis because of the strict inclusion criteria. Secondly, the analysis’ findings were derived from primary studies on speaking abilities and listening comprehension. These findings need to be interpreted cautiously to infer videoconferencing’s effect on overall L2 abilities. Next, the publication bias found in the analysis appears to adversely affect the estimation of the effect of videoconferencing. Lastly, this meta-analysis included only one study that targeted listening skills, which suggests that the results regarding this range of skills have to be treated with caution. To minimize the limitations, some suggestions are provided for future studies. Firstly, fugitive literature (i.e., unpublished work, such as dissertations/theses, papers presented in meetings, and technical reports [74]) can be included to deal with publication bias and increase the number of studies for analysis. Secondly, videoconferencing studies with a single-group design (within-group comparison) can be considered for inclusion in the analysis. Thus, a comprehensive picture of the effectiveness of videoconferencing on L2 learning can be exhibited.

## Figures and Tables

**Table 1:** Systematic reviews and meta-analyses of computer-mediated communication for SLA purposes.

Study	Lin [14]	Lin [15]	Lin et al. [16]	Sauro [23]
Research Focus	Computer-mediated communication	Computer-mediated communication	Synchronous text-based computer-mediated communication	Synchronous computer-mediated communication
Types of Studies Included	Journal articles, dissertations, theses, technical reports	Journal articles, dissertations, theses, technical reports	Journal articles and dissertations	Journal articles
The Publication-Year Duration of Included Studies	2000–2012	2000–2012	1990–2012	1990–2010
Number of Analyzed Studies	59	59	10	97

**Table 2:** Coding scheme (adapted from Lin et al. [16]).

Feature	Name of Features	Definition of the Features
1	Study ID	Assign an identification number to each study
2	Author	First name + last name
3	Publication year	The publication year
4	Participants’ L2-proficiency level	Participant L2 proficiency level, e.g., low, mid, and high levels
5	Participants’ education level	Participant educational background. e.g., college level
6	Learning context	Participant learning environment, e.g., ESL, EFL, FL, SL
7	First language (L1)	The participants’ mother tongue
8	Target language (TL)	The L2 examined in the study
9	Independent variable (IV) (intervention)	The intervention given to participants in the study, e.g., using SCMC tools to complete tasks
10	SCMC tool	The SCMC software used in the study
11	SCMC activity	The activities in the study. e.g., jigsaw, information-gap, discussion
12	Treatment length	The time spent doing each SCMC task
13	Treatment duration	The duration of undergoing treatment for participants
14	Dependent variable (DV) (TL measures)	The variable to measure or assess the effects of an independent variable, e.g., test scores and ratings
15	Target language features	The language aspect examined. e.g., lexical development, oral performance, grammatical competence
16	Pretest	The type of pretest and measurement
17	Post-test	The type of post-test and measurement
18	Delayed post-test	The type of delayed post-test and measurement
19	Sample size	The total sample size of this study
20	Research design ^a^	1. Between subjects (with a comparison group) 2. Between subjects (with a true control group) 3. Between subjects (with a true control group and at least one comparison group)
21	Experimental group (EG)	Videoconferencing software program
22	Comparison group (CG)/true control group (TG)	Learning mode assignment, e.g., another type of CMC group, face-to-face group, true control group (no intervention)
23	Data collected for analysis	The data collected for analysis in the study, e.g., test scores, questionnaires, chat-log transcripts

^a^ Comparison groups refer to a face-to-face communication group, an asynchronous CMC communication group, or a synchronous communication CMC group other than video-based mode, while a true control group means no involvement in interaction or communication activities at all.

**Table 3:** Summary of key features of the selected five studies.

	*N*	Target Language	Learning Context	L2 Proficiency	Language Features	Treatment Duration	SCMC Activity Length	Group Division	Comparison/Control Groups
Alshahrani [23]	36	English	EFL	pre-intermediate to intermediate	Speaking performance	12 weeks	1 h (two times per week)	A whole group	Face-to-face interaction
Rassaei [39]	57	English	EFL	intermediate	Speaking performance	10 days	14 min per time	One-on-one	Face-to-face interaction and no interaction
Saito and Akiyama [6]	30	English	EFL	Not reported	Speaking performance	10 weeks	1 h (30 min in English; 30 min in Japanese) per time	One-on-one	No interaction
Saito and Akiyama [26]	30	English	EFL	Not reported	Listening comprehension	10 weeks	1 h (30 min in English; 30 min in Japanese) per time	One-on-one	No interaction
Tecedor and Campos-Dintrans [25]	48	Spanish	SFL	beginning	Speaking performance	12 weeks	50 min (3 times per week)	Pair	Face-to-face interaction and asynchronous voice recording

**Table 4:** Effect sizes on immediate post-tests.

Study	Effect Size (Hedge’s *g*)	Standard Error	Confidence Interval for Effect Size		*p*	Heterogeneity		
			Lower	Upper		Q-Value	df	*I* ^2^
Alshahrani [23]	0.29	0.33	−0.35	0.94	0.37	2.83	4	0.00
Rassaei [39]	0.11	0.31	−0.51	0.73	0.73			
Saito and Akiyama [6]	0.82	0.37	0.09	1.54	0.03 *			
Saito and Akiyama [26]	0.54	0.36	−0.17	1.25	0.13			
Tecedor and Campos-Dintrans [25]	0.16	0.33	−0.48	0.80	0.63			
All studies	0.35	0.15	0.06	0.65	0.02 *			

* *p* < 0.05.

## Data Availability

The datasets generated and/or analyzed during the current study are available from the corresponding author on reasonable request.
